# A Pilot Proteomic Analysis of Huntington’s Disease by Functional Capacity

**DOI:** 10.3390/brainsci15010076

**Published:** 2025-01-16

**Authors:** Andrew McGarry, Ruin Moaddel

**Affiliations:** 1 Cooper University Healthcare at Rowan University, Camden, NJ 08103, USA; 2 Biomedical Research Center, National Institute on Aging, National Institutes of Health, Baltimore, MD 21224, USA

**Keywords:** HD, cerebrospinal fluid, inflammation, NAD^+^ metabolism, TFC score

## Abstract

**Background:** The molecular biology of Huntington’s Disease (HD) has grown substantially, with pathological considerations extending to genetic modifiers, epigenetic changes, transcriptomics, the proteome, and the metabolome. The metabolome and proteome are especially intriguing in that they most directly reflect the functional state of the cellular environment, which may involve some combination of pathology as well as compensation. **Methods:** We assessed CSF proteomics from eight participants by their functional severity (TFC range 3–13), with 47 proteins having a minimum r-value of 0.7 and nominal *p*-values < 0.05. **Results**: Our exploratory data reveal correlations between progression and several processes including inflammation, ECM homeostasis and NAD^+^ metabolism. **Conclusions:** Consistently identified targets that correlate with phenotype or progression may have value, if validated, as enrichment tools in clinical trials and potentially as markers of therapeutic response.

## 1. Introduction

Since identification of its causative gene in 1993 by the Huntington’s Study Group, the understanding of Huntington’s Disease (HD) molecular biology has expanded substantially, with pathological considerations extending to genetic modifiers, epigenetic changes, transcriptomics, the proteome, and the metabolome [1]. These biological features beyond the *htt* gene defect may offer opportunities to explore potential therapeutic targets or predictive biological signatures for particular clinical features or progression. The metabolome and proteome are especially intriguing for these considerations in that they most directly reflect the functional state of the cellular environment, which may involve some combination of pathology as well as compensation. Thus, the metabolome and proteome can be viewed as the fundamental output of a complex system with direct consequences for homeostasis and survival. They may also offer practical advantages from an experimental therapeutics or biomarker point of view, in that manipulation of metabolites or proteins may be simpler to design and test therapies for and more accessible for monitoring progression or response to an intervention if validated as useful for these purposes. Accordingly, further study of the metabolome and proteome in HD is warranted.

There is limited literature on the CSF proteome in HD. Fang and colleagues assayed 20 participants categorized by UHDRS Independence Score (IS) as either early (>80, N = 10) or moderate-stage (65< x <80, N = 10) or controls [2,3] and saw increases in numerous proteins associated with immune system function, while brain-specific proteins decreased. A study of nine HD participants averaging a TFC of 9.2 +/− 1.2 (range 3–13) showed prothrombin, ApoA4, and haptoglobin increasing in HD compared to controls [4]. Niemela et al. examined 12 manifest and 13 premanifest HD patients with controls and found numerous differences between HD and pre-manifest HD, including more upregulated than downregulated proteins (43 vs. 10) [5]. Another study of 16 manifest and 8 premanifest HD patients with 8 controls demonstrated a range of protein levels correlating to severity in manifest HD as measured by TFC grouping (early/mid-stage, TFC > 5 and late-stage, TFC < 5) [6].

We previously examined the HD metabolome cross-sectionally in plasma and CSF for participants with varying degrees of functional impairment [7]. Here, we extend that analysis and describe the CSF proteome from these participants according to their functional severity. The purpose of this analysis was to explore CSF proteome changes in manifest HD using a broad protein panel and wide-ranging individual TFC scores.

## 2. Methods

### 2.1. Participant Selection

We previously reported on the plasma and CSF metabolomic profiles for 12 participants with manifest HD of varying severities according to their Total Functional Capacity Score [8]. This is a 13-point scale that assesses functional performance in 5 domains: capacity for work (3 = normal; 2 = reduced capacity; 1 = marginal work; 0 = unable), finances (3 = normal; 2 = slight assistance; 1 = major assistance; 0 = unable), domestic chores (2 = normal; 1 = impaired; 0 = unable), activities of daily living (3 = normal; 2 = minimal impairment; 1 = gross tasks only; 0 = total care), and care level required (2 = home; 1 = home or chronic care; 0 = full time skilled nursing). Scores range from 13 (normal) to 0 (total incapacitation) and define stages of the disease: HD1 (TFC 11–13), HD2 (TFC 7–10), HD3 (TFC 3–6), HD4 (1–2), and HD5 (0). Of the 12 participants, 8 contributed sufficient quantities of CSF for proteomic analysis. Table 1 shows demographics for these participants. Two were male and six were female, with ages ranging from 30 to 57 (mean 48 years). TFC scores for these participants ranged from 3 to 13 (3, 4, 5, 6, 7, 8, 12, 13).

### 2.2. Statistical and String Analyses

**Figure 1:**
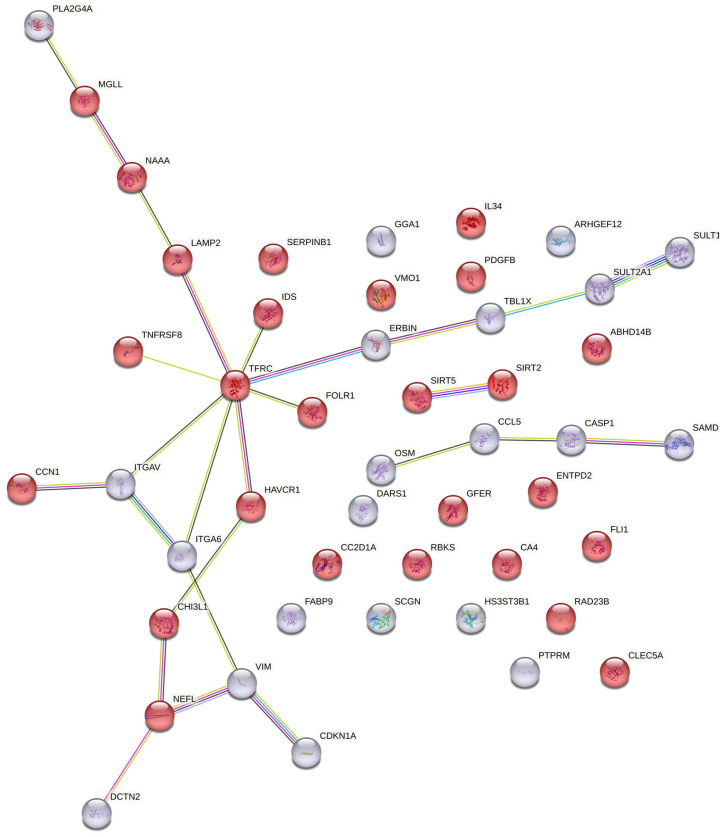
STRING analysis for proteins with r-value correlations +/− 0.7 to 1.0 and nominal *p*-values < 0.05 for proteins above LOD (red, n = 26) and below LOD (gray, n = 21) using the full STRING network, active interaction sources (Textmining, experiments, databases, co-expression, neighborhood), medium confidence (0.400).

Pearson correlation coefficients were generated for each protein, with a minimum r-value of 0.7 (strong correlation) for further consideration. Comparisons between groups were exploratory and unadjusted for multiplicity; all *p*-values are nominal. To gain broader insight into the molecular mechanisms of progression, proteins that were nominally different (*p* < 0.05) were further analyzed. String analysis was carried out on proteins that were above the level of detection (LOD) (Figure 1) and all proteins separately to identify central hubs for each maximal timepoint with the full string network, active interaction sources (Textmining, experiments, databases, co-expression, neighborhood), and medium confidence (0.400).

### 2.3. Protein Measurement

CSF proteins were measured using the commercially available Olink Explore 1536 kit (Olink Proteomics AB, Uppsala, Sweden) according to the manufacturer’s instructions (www.olink.com) and as previously described [8]. This kit measures a total of 1472 unique proteins. The raw output data are quality controlled, normalized, and converted into Normalized Protein eXpression (NPX) values (Table S1). All assay validation data are available on the manufacturer’s website. Due to the exploratory nature of the study, limited sample size and use of CSF matrix, all proteins measured including those below the level of detection (>62.5%, or 5/8 below LOD) were considered.

## 3. Results

In total, 47 proteins had a minimum r-value of 0.7 and nominal *p*-values < 0.05, with 26 of these being classified above the level of detection (above LOD for at least three participants) and 21 below (Table 2). Most proteins increased with progression (i.e., negative correlation with functional decline).

Enrichment analysis of all proteins (above and below LOD) identified extracellular exosomes (cellular component GO), positive regulation of PI3K signaling (KEGG pathway), cellular response to cytokine stimulus (biological process GO), and neurodegeneration (Human Phenotype Ontologies) as the top hits using EnrichR (https://maayanlab.cloud/Enrichr/, accessed on 10 December 2024) [9,10,11]. The top identified KEGG pathways for proteins above the LOD were related to NAD^+^ metabolism (KEGG and Wiki Pathways, GO Molecular Function). String analysis was carried out on the proteins that had r values greater than ǀ±0.7ǀ and *p*-value < 0.05 to identify central hubs for all proteins (above and below LOD) with the full string network, active interaction sources (Textmining, experiments, databases, co-expression, neighborhood), and medium confidence (0.400) (Figure 1). Four proteins had greater than three interactions, including VIM, NEFL, ITGAV and TFRC, with only VIM below the LOD (Figure 1).

## 4. Discussion

This analysis of CSF from eight Huntington’s Disease participants of varying severity suggests potential insights for proteomics of the neurodegenerative process in relation to functional decline. Our exploratory data reveal correlations between progression and regulators of inflammation, ECM proteins and NAD^+^ metabolism. Several of the identified proteins are related to oxidative stress, calcium homeostasis, DNA damage responses, the cell cycle, regulation of the interstitial space, and apoptosis.

Observed increases in inflammatory markers and immune-related processes are consistent with suspected pathophysiology in HD [12,13]. Concentrations of the pro-inflammatory cytokine IL-34, known to activate CNS microglia, increased with functional decline (Table 1) [14]. Inhibition of the IL-34 receptor in rodent CNS reduced the number of microglia and ameliorated mHTTx1-mediated neurodegeneration [15]. IDS is thought to play a role in the maintenance of cytokine and chemokine levels [16]. TNFRSF8 (CD30), which decreased with progression, regulates T-cell differentiation and gene expression through activation of NF-kB, a pro-survival regulator of apoptosis [17]. How reduction in CD30 with progression relates to overactivation of NF-kB in HD, which has been identified in cell cultures, murine models, and astrocytes from the human HD brain, is unclear [18]. Immune-related Increases included CLEC5a, implicated in septic and aseptic inflammation [19]; platelet-derived growth factor B-subunit (PDGFB), a regulator of astrocyte function for which homo- and heterodimers exhibit a range of protective and deleterious effects in the CNS [20]; and SERPINB1, a serine protease inhibitor that protects tissues from inflammatory damage [21]. Some of these inflammatory proteins do not have well-defined roles in HD at this time but may deserve further study.

Other proteins increasing with progression include CC2D1A, a calcium-dependent transcriptional repressor of the serotonin receptor (HTR1A), and neurofilament light chain (NEFL), which has been observed elsewhere to increase in HD plasma and CSF with progression [6,22,23,24]. LAMP2, a regulator of lysosomal pH and chaperone-mediated autophagy, also increased [25]. Recent postmortem analyses of HD brains across varying stages (HD 2–4) show increased striatal expression of LAMP1 and LAMP2 mRNA earlier in progression, which is suggestive of a compensatory response to account for increasing mHtt aggregates [26]. CA4 is expressed on the luminal surface of the capillary endothelium and serves as a marker of the blood–brain barrier [27]. Degradation of the blood–brain barrier is observed in HD and HD models [28,29]. The increase in CA4 with disease progression may simply reflect greater vascular density, as increased vascular densities have been observed in the cortex, striatum, and substantia nigra in HD patients and R6/2 mice [30]. Since CA4 is integral in the maintenance of intracellular and extracellular pH, the increase may represent an attempt to manage acid–base balance in an increasingly anaerobic environment with accumulation of lactate [31,32]. Brain pH is higher in HD compared to healthy controls as measured by 31-P magnetic resonance spectroscopy [33].

We have previously described urea cycle dysregulation (elevated arginine, citrulline, ornithine) and increasing CSF NAD^+^ levels with progression in this cohort [7]. In the present proteomic analysis, NAD^+^ metabolism was the top identified pathway in the same participants. CSF levels of the NAD^+^-dependent deacetylases, SIRT2 (cytosolic) and SIRT5 (mitochondrial), both increased with progression. The therapeutic potential of sirtuins in HD has been reviewed [34]. SIRT2 inhibition may improve impaired cholesterol biosynthesis and lessen mHtt aggregation in HD [35]. While an association of SIRT5 with progression in HD has not previously been reported, its roles in regulating glycolysis, fatty acid metabolism, nitrogenous waste, regulation of cellular homeostasis, and protecting against mitochondrial dysfunction would be consistent with the influence on progression in HD [36]. SIRT5 promotes anti-oxidative defenses in mitochondria by upregulating IDH2, G6PD, and SOD 1 (r = −0.536) [37,38,39]. SIRT5 also upregulates carbamoyl phosphate synthetase 1, the initial step in the urea cycle [40]. Whether SIRT5 upregulation may be promoting increased conversion of excessive arginine into urea, a toxic metabolite known to accumulate in the human HD brain, is unknown [41]. SIRT5 would appear to be advantageous to upregulate therapeutically, but any indirect deleterious effect on urea could be a potential consideration in the design of such an agent. Our previous longitudinal observation suggesting reduced AMPK activity, a change expected to upregulate SIRT5 expression, is consistent with the current data [42,43].

Several proteins below the LOD had strong inverse correlations with TFC and are involved in pathways similar to those identified above, including vimentin (VIM), CCL5 and CDKN1A. VIM, an intermediate filament protein suggested to promote mutant Htt toxicity by altering IRBIT/IP3R1 biology and subsequent calcium homeostasis/aggresome formation, increased with progression [44]. Inhibition of Rho kinase, which phosphorylates and activates vimentin, has been investigated in murine models of HD [45]. CCL5 binds to CCR5, a G-protein coupled receptor highly expressed in microglia with lesser levels in astrocytes and neurons [46]. Once activated by CCL5, upregulated neuronal CCR5 impairs the clearance of several autophagy substrates, including polyglutamine aggregates, through the well-described PI3K-AKT-mTORC1 pathway [47]. The increase in CCL5 with progression suggests CCR5 may play a role in HD and is consistent with observations in a pre-manifest HD mouse model, where CCR5 is also increased [47]. The CCR5 antagonist maraviroc improves mTORC1-mediated autophagy dysregulation and lessened accumulation of mutant huntingtin in mouse models [47].

CDKN1A (cyclin-dependent kinase inhibitor 1a, p21) also increased with progression. CDKN1A binds cyclin–cyclin-dependent kinase 2 or cyclin-dependent kinase 4, regulating the cell cycle and its relation to transcription and DNA repair; its expression is closely controlled by p53, and the means with which cell cycle arrest may occur in G1 in response to stress [48,49]. CDKN1A is a substrate of caspase-3 and can have a prominent role in inhibiting apoptosis [50]. Increases in HD may reflect attempts to regulate apoptosis, an attempt at DNA repair, or some combination; interest in DNA repair loci as modifiers of onset and progression has grown, in light of polymorphisms in repair-associated loci [51].

Only three proteins (FLI1, TNFRSF8 and SCGN) decreased with progression. Secretagogin (SCGN), a calcium-binding protein found in the human cerebellum, hippocampus, hypothalamus, and striatum, is thought to modify the release of stress hormones including corticotropin-releasing hormone (CRH) and ACTH, influence adrenergic tone and cortical excitability, and act as a calcium sensor [52,53]. Little is known regarding SCGN biology in HD. Reduction in SCGN with progression may simply reflect general degeneration and overall interneuron loss, though it is interesting to consider if relative effects on interneurons in direct and indirect pathways may be more nuanced [53]. Given the neuroprotective role that calcium-binding proteins are thought to play (tiers of substantia nigra, for example), whether SCGN has a similar protective quality and whether that influences striatal output with progression are currently unknown. Preferential excitotoxicity in indirect pathway neurons due to lost regulation by more vulnerable SCGN interneurons may promote earlier demise and unopposed direct pathway output, perhaps contributing to hyperkinetic movements in HD.

In comparison to existing HD CSF proteome literature, we also observed an increase in NEFL with progression, a moderate increase in the cell surface proteoglycan GPC1 that did not meet the threshold (r = −0.512), and a moderate trend toward an increase with progression in C1Qa (−0.4511), a component of the C1Q molecule that has been implicated in HD pathogenesis and for which the associated protein C1Qb has been reported to correlate with disease severity [5,6,54]. We note these consistencies with previously reported studies and also that methodological differences in what proteins were assayed and how clinical severity was characterized may account for differences between the present data and other studies’ observations on change within manifest disease. A number of our highlighted findings have not been reported elsewhere in human HD CSF reports, which may also reflect differences in methodology.

These data are exploratory and hypothesis-generating only. Interpretations are limited by the small sample size (n = 8) and concentrations of several analytes with relative values that were below the level of detection despite fairly strong correlations, potential biological plausibility, and nominal *p*-values. The relationships identified here need to be replicated in a larger study. No adjustments for multiplicity were made, and so all *p*-values are nominal. Data may reflect some element of peripheral metabolism secondary to a disrupted blood–brain barrier, and CSF has inherent limitations in reflecting intraparenchymal conditions. As with our plasma analysis, clinical progression may reflect other non-HD influences on function, and concomitant medications may influence the proteome in some way.

## 5. Conclusions

In HD CSF samples from participants of wide-ranging functional disability, we observed correlations between progression and several processes including inflammation, ECM protein homeostasis, and NAD^+^ metabolism. Future work will look to prospectively assess the proteome and metabolome in a larger longitudinal sample, including controls, towards the identification of a reliable biological signature of severity or progression. Consistently identified targets that correlate with phenotype or progression may have value, if validated, as enrichment tools in clinical trials and potentially as markers of therapeutic response.

## Figures and Tables

**Table 1:** Participant demographic data, taken from [7].

Participant	Age at Enrollment	Gender	Year of Diagnosis	CAG Expanded Repeat Length	Baseline TFC	HD Stage
2	55	M	2012	N/A	4	III
3	55	F	2014	43	7	II
4	45	F	2011	N/A	6	III
5	42	F	2014	N/A	3	IV
7	52	F	2011	42	12	I
8	46	M	2013	45	5	III
9	57	F	2015	43	8	II
10	30	F	2017	43	13	I

N/A: For CAG repeat length, diagnosis was established by first-degree relative and diagnostic confidence level IV.

**Table 2:** Correlations between proteins in CSF and functional progression (TFC score) are presented. Only r-value correlations of +/− 0.7 to 1.0 with nominal *p*-values < 0.05 are shown, with proteins that were above detection in at least 3 participants (**A**) and below detection for >5 participants (**B**).

**A**		
**Negative Correlations**	**r-Value**	***p*-Value**
CLEC5A	−0.9172	0.0013
IDS	−0.8808	0.0039
CA4	−0.8786	0.0041
NAAA	−0.8360	0.0097
CC2D1A	−0.8045	0.016
FOLR1	−0.7935	0.0188
ABHD14B	−0.7917	0.0192
MGLL	−0.7876	0.0203
SERPINB1	−0.7868	0.0205
NEFL	−0.7756	0.0237
SIRT5	−0.7654	0.0269
RAD23B	−0.7645	0.0271
GFER	−0.7638	0.0274
IL34	−0.7633	0.0275
CHI3L1	−0.7548	0.0304
LAMP2	−0.7534	0.0309
TFRC	−0.7440	0.0343
PDGFB	−0.7218	0.0432
SIRT2	−0.7205	0.0438
CCN1	−0.7181	0.0448
VMO1	−0.7157	0.0459
ENTPD2	−0.7138	0.0467
RBKS	−0.7118	0.0476
HAVCR1	−0.7075	0.0496
**Positive Correlations**		
FLI1	0.7786	0.0228
TNFRSF8	0.8216	0.0124
**B**		
**Negative Correlations**	**r-Value**	***p*-Value**
CCL5	−0.8740	0.0045
ARHGEF12	−0.8730	0.0046
FABP9	−0.8656	0.0055
TBL1X	−0.8174	0.0132
CDKN1A	−0.7896	0.0198
OSM	−0.7729	0.0245
SAMD9L	−0.7659	0.0267
DCTN2	−0.7612	0.0282
VIM	−0.7595	0.0288
ITGA6	−0.7362	0.0373
ITGAV	−0.7318	0.039
SULT1A1	−0.7300	0.0398
PTPRM	−0.7289	0.0402
CASP1	−0.7241	0.0422
SULT2A1	−0.7239	0.0423
ERBIN	−0.7224	0.043
GGA1	−0.7192	0.0444
DARS1	−0.7191	0.0444
HS3ST3B1	−0.7157	0.0459
PLA2G4A	−0.7097	0.0486
**Positive Correlations**		
SCGN	0.8770	0.0042

## Data Availability

The raw data supporting the conclusions of this article will be made available by the authors on request.

## Supplementary Materials

The following supporting information can be downloaded at https://www.mdpi.com/article/10.3390/brainsci15010076/s1: Table S1: Panel of proteins measured in the CSF of participants with HD (from Table 1) with the reported Total Functional Capacity (TFC) and the corresponding measured limit of detection (LOD) and Normalized Protein eXpression (NPX) values.
